# A Lightweight and High-Precision PCB Surface Defect Detection Method Based on YOLOv8

**DOI:** 10.3390/jimaging12060266

**Published:** 2026-06-18

**Authors:** Zhenling Wang, Ya Gao, Ying Xiao, Qiurui He

**Affiliations:** 1 School of Internet of Things Engineering, Wuxi Institute of Technology, Wuxi 214121, China; gaoya@wxit.edu.cn (Y.G.); xiaoy@wxit.edu.cn (Y.X.); 2 School of Software, Luoyang Normal University, Luoyang 471934, China; heqiurui@lynu.edu.cn

**Keywords:** PCB surface defect detection, YOLOv8, high precision, lightweight

## Abstract

In response to the diverse types and large number of PCB surface defects, our paper proposes an improved YOLOv8-based method for PCB surface defect detection. First, a lightweight modification is performed by introducing RepGhostBottleNeck as the lightweight backbone network, which reduces the number of parameters in the training model. It should be noted that the term “lightweight” in this paper is relative to the original YOLOv8L baseline. Compared with extremely lightweight detectors, the model in this paper places greater emphasis on the balance between accuracy and efficiency. Additionally, an attention mechanism module and a small object detection head module are added to the backbone network. Furthermore, the loss function of the network is improved. Experimental results show that the improved model achieves an average mAP@0.5 of 0.976, demonstrating high-precision detection on the constructed dataset.

## 1. Introduction 

With the continuous development of electronic technology, the assembly of PCB also changes. The number of electronic components such as resistors, capacitors, and integrated circuits on printed circuit PCBs has been increasing, leading to the development of miniaturization, integration, and diversification of PCB boards. Surface Mount Technology (SMT) has enabled the high-density and high-speed automatic assembly of components. The development of these technologies has brought challenges to PCB inspection technology. Conducting fast and accurate detection and identification of defects on PCB has become key to improving PCB quality.

PCB components vary greatly in type, appearance, and size, making manual inspection methods based on human vision and electrical testing [1] unable to meet the demands of automated production. Machine vision (MV) methods based on image processing have also been widely used for various PCB defect detection tasks [2,3,4,5,6]. These machine vision methods have strict requirements for size matching between the PCB board and the inspected image, which is relatively difficult to achieve. Additionally, the detection efficiency of these methods is closely related to the quality of the images, and they are typically used for detecting single defects; hence the actual defect detection rate is relatively low.

As the electronic manufacturing industry advances toward high-density and high-integration development, PCB surface defect detection is required not only to meet detection accuracy requirements but also to comply with industrial quality control standards. According to PCB quality acceptance standards such as GOST R 56251-2014 and IPC-A-600, defects including open circuits, short circuits, missing holes, mouse bites, spurs, and spurious copper may affect circuit connection reliability and product performance. Therefore, these defects are regarded as critical defects that require focused inspection during PCB production.

In recent years, deep learning-based object detection methods have been widely applied in the field of PCB defect detection. The YOLO series of models has attracted considerable attention due to its high detection accuracy and real-time performance. However, existing YOLO models still face challenges such as a large number of parameters, insufficient detection capability for small target defects, and high industrial deployment costs. To address these issues, researchers have proposed methods including GhostNet [7], coordinate attention [8], lightweight detection heads, and improved bounding box loss functions. Nevertheless, the comprehensive application of these methods in complex PCB defect scenarios still requires further research.

Recently, the performance of computers is continuously enhancing. Machine learning techniques, including Deep Learning (DL) [9] and Convolutional Neural Networks (CNNs), have been widely applied in the field of PCB defect detection [10,11]. Additionally, the Region-based Convolutional Neural Network (R-CNN) [12,13] and similar networks [14,15] have been introduced subsequently and applied to PCB surface defect detection. These networks achieve object detection through two steps: they extract object regions and then classify and recognize these regions using CNNs. These aforementioned methods leverage the inherent multi-scale and pyramidal hierarchy of deep convolutional networks to achieve high detection accuracy, but the two-stage detection limits the detection speed. It is worth noting that there are a large number of tiny targets in PCB defects (e.g., mouse bites, burrs, etc.), whose pixel proportion is typically less than 0.01%, posing a serious challenge for detection. Systematic reviews by Tong et al. [16] and Liu et al. [17] indicate that the core difficulty in small object detection lies in the easy loss of small target information in deep feature maps, along with insufficient semantic information in shallow feature maps. To address this issue, this paper introduces a P2 small object detection head into YOLOv8, fusing high-resolution shallow features with deep semantic features to improve the detection accuracy of tiny defects. To address the issue that two-stage models are too slow for real-time detection, single-stage networks such as the YOLO series [18,19] directly classify and regress on the input, and are widely applied in industrial detection [20].

### 1.1. Motivation and Contributions

This paper addresses the challenges of small target detection difficulty, high model complexity, and insufficient industrial deployment efficiency in PCB surface defect detection tasks by collaboratively optimizing the lightweight design and detection performance of YOLOv8L [21]. The main contributions are as follows:

- The RepGhostBottleneck is used to replace the Bottleneck structure in the original YOLOv8 backbone network, significantly reducing the number of model parameters and computational complexity while maintaining feature extraction capability;
- The Coordinate Attention (CA) mechanism is introduced to enhance the model’s ability to represent fine-grained defect features and positional information;
- A P2 small target detection head is added to improve the model’s detection capability for tiny PCB defects;
- The WIoU loss function is adopted to optimize the bounding box regression process, thereby improving target localization accuracy and model generalization ability;
- The effectiveness of the proposed method is validated on a hybrid dataset constructed from a public PCB defect dataset and an actual industrial dataset, achieving a synergistic improvement in detection accuracy and inference efficiency.

It should be noted that the term “lightweight” in this paper is relative to the original YOLOv8L baseline (43.7 M parameters). The final model in this paper has 34.0 M parameters, representing a reduction of approximately 22%. Compared with extremely lightweight detectors (e.g., PCB-YOLO with 2.3 M parameters), our model places greater emphasis on achieving a favorable balance between detection accuracy and model complexity, making it suitable for industrial inspection scenarios that demand high accuracy.

### 1.2. Organization

The remaining part of our paper is organized as follows. The YOLOv8 model and improvements are presented in Section 1. In Section 2, data preparation and experimental configuration are described. Furthermore, experimental validation is provided in Section 3. Finally, the conclusions of the paper are presented in Section 4.

## 2. YOLOv8 Model and Improvements

### 2.1. YOLOv8 Detection Model

In this paper, the YOLOv8 detection model [20] is further improved. Next, we provide a detailed introduction to the backbone, neck, and prediction head of YOLOv8, as well as their respective improvements.

#### 2.1.1. Backbone Network

The backbone network of YOLOv8 also draws on the structure of CSPDarkNet [21]. While maintaining feature extraction capability, CSPDarkNet optimizes information flow and feature reuse, thereby improving the training efficiency of the model. The biggest difference between YOLOv8 and YOLOv5 is that the C2f module replaces the C3 module. The C2f module reduces redundant parameters through its efficient structural design, thereby enhancing both computational efficiency and feature extraction capability. In addition, the Backbone also incorporates common improvement techniques such as Depthwise Separable Convolution and Dilated Convolution to enhance feature extraction capability. The final layer of the Backbone passes through SPPF (Spatial Pyramid Pooling Fast), which helps expand the receptive field while reducing computational cost. Through these designs, the YOLOv8 backbone network achieves efficient and powerful feature extraction capability, providing a solid foundation for subsequent object detection tasks.

#### 2.1.2. Neck

The neck network of YOLOv8 is located between the backbone network and the head network. Through feature enhancement techniques such as Feature Pyramid Network (FPN) or Path Aggregation Network (PANet), the neck network significantly improves the representational power of features, enabling the model to better recognize objects. Some neck network designs, such as SPPF (Spatial Pyramid Pooling Fast), can enhance model performance without significantly increasing computational cost.

#### 2.1.3. Prediction Head

The prediction head is a key component in an object detection model. Its main task is to utilize feature information passed from the backbone network and neck network to predict the location, size, and category of objects within an image. The prediction head determines the precise position and dimensions of objects in the image through regression analysis. It is also responsible for classifying the object within each bounding box, determining which predefined category the object belongs to. In addition to location and category, the prediction head assigns a confidence score to each predicted bounding box, indicating the probability that the bounding box contains an object. The design and implementation of the prediction head are crucial to the performance of an object detection model, as it needs to strike a balance between accuracy and speed to accommodate different application scenarios.

### 2.2. Enhancement of the YOLOv8 Detection Model

#### 2.2.1. Enhancement of the Backbone Feature Extraction Network

GhostNet is a lightweight network model first proposed by Han et al. [7] at CVPR 2020. Its core idea is to generate redundant feature maps through cheap operations, enabling feature extraction while preserving the original convolutional features, thereby reducing the computational cost and number of parameters of the network.

**Figure 1:**
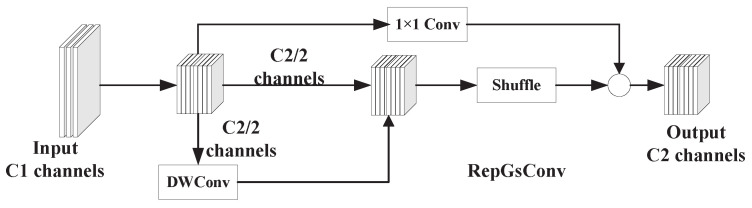
RepGhostConv Structure.

Our paper modifies GhostNet with an operation named RepGhostConv. As shown in Figure 1, a regular convolution is first applied to transform the input image C1, producing an output with C2 channels. The C2 channels are then grouped, where C2/2 channels are processed using DepthWise convolution (DW convolution). The output is then concatenated with the other C2/2 feature maps, followed by a channel shuffle operation. Meanwhile, a 1×1 convolution is applied to the C2 channels to enhance the channels and increase the network’s nonlinearity. The output of the 1×1 convolution is then added to the concatenated feature maps, resulting in a feature map with C2 output channels.

**Figure 2:**
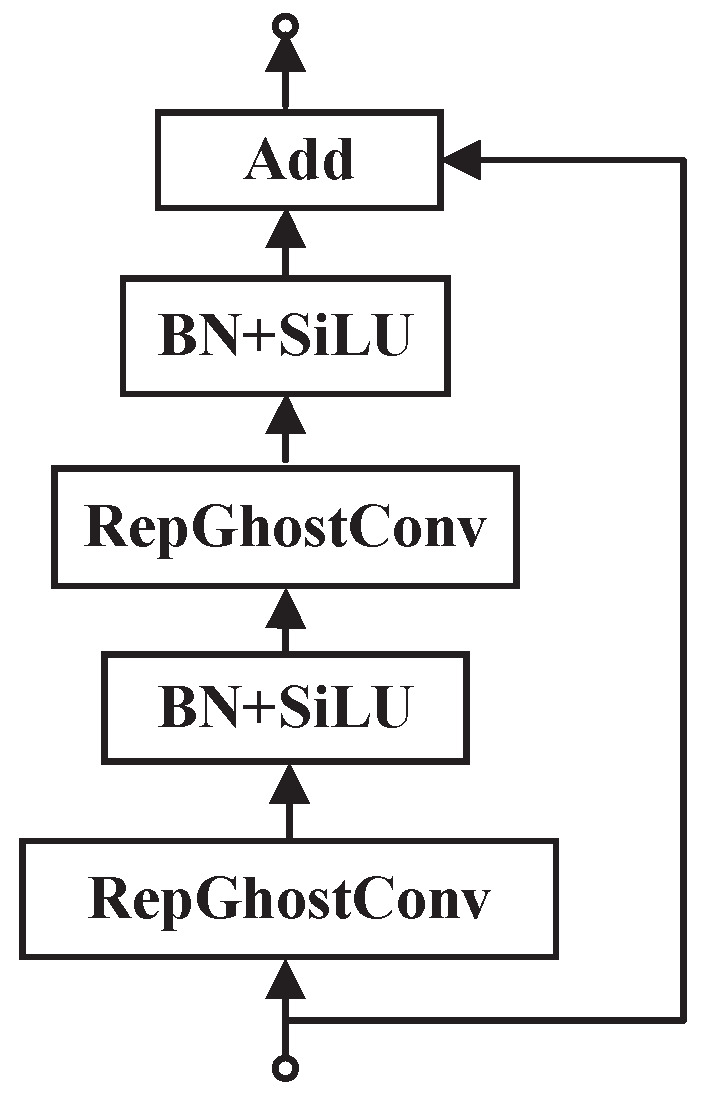
RepGhostBottleNeck Network Structure.

As shown in Figure 2, RepGhostBottleneck first passes through the first RepGhostConv to increase the number of channels, followed by normalization and the SiLU activation function. It then passes through the second RepGhostConv to reduce the number of channels of the output feature map, matching the number of input channels. Finally, the feature map obtained from the previous step is added to the residual connection for feature fusion.

This paper replaces all Bottleneck modules in the C2f module with RepGhostBottleneck. The feature fusion strategy in this structure enhances the variability of feature learning between different network layers, thereby improving the network’s learning capability. Additionally, the use of depthwise convolution (DW convolution) in the module significantly compresses the model size, reducing the number of parameters and accelerating the speed of forward inference.

#### 2.2.2. Coordinate Attention (CA) Mechanism

**Figure 3:**
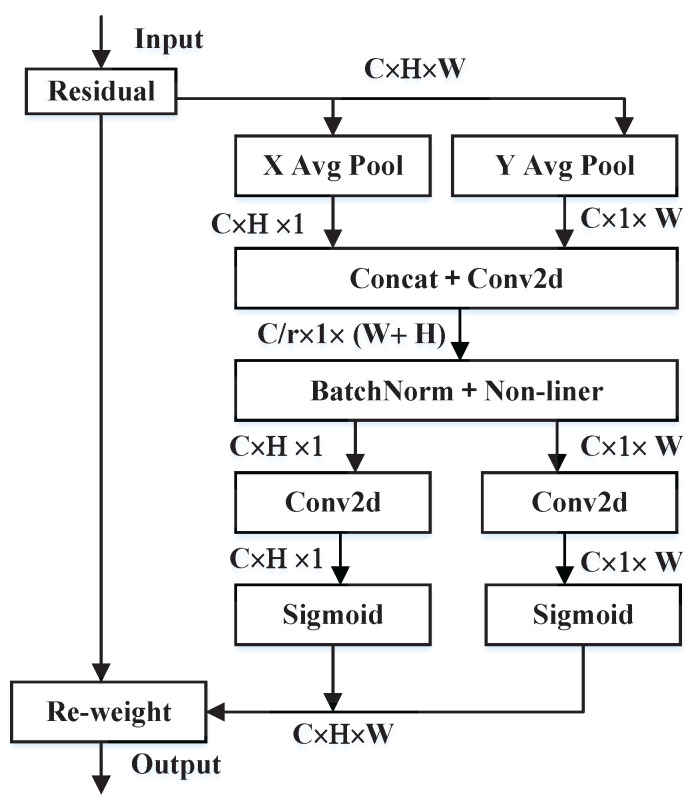
CA Coordinate Attention Mechanism.

To detect small defects on PCB boards, the CA coordinate attention mechanism is added after the C2f layer to enhance the learning capability of the deep network. The CA coordinate attention mechanism was proposed by Hou et al. [8] at CVPR 2021. The CA mechanism decomposes channel attention into two one-dimensional feature encoding processes that aggregate features along two different directions (horizontal and vertical). The model can independently capture long-range dependencies along one spatial direction while preserving precise positional information along the other spatial direction. By incorporating positional information into channel attention and encoding features from different directions, the CA mechanism enhances the model’s feature representation capability, thereby improving its learning ability, as shown in Figure 3.

For a given input *X*, each channel is encoded along the horizontal and vertical coordinates using ranges of $\left(H,1\right)$ and $\left(1,W\right)$, respectively. *H* and *W* represent the height and width of the input feature tensor *X* respectively. Therefore, the output of the *c*th channel at the height *h* can be expressed as follows:

(1) $$\begin{array}{c} z_{c}^{h}\left(h\right)=\frac{1}{W}\sum_{0\leq i\leq W}x_{c}\left(h,i\right), \end{array}$$

where *W* is the channel width feature vector and $x_{c}$ is the input value of the *c*th channel.

Similarly, the output expression of the *c*th channel at width *w* can be obtained as shown in Equation (2):

(2) $$\begin{array}{c} z_{c}^{w}\left(w\right)=\frac{1}{H}\sum_{0\leq j\leq H}x_{c}\left(j,w\right), \end{array}$$

where *H* represents the feature vector of the channel height.

After embedding the aforementioned information, the generated aggregated feature maps are connected, and then transformed by the 1×1 convolutional transformation function $F_{1}$, as shown in Equation (3):

(3) $$\begin{array}{c} f=\delta \left(F_{1}\left(\left[z^{h},z^{w}\right]\right)\right), \end{array}$$

where $\left[z^{h},z^{w}\right]$ represents the connection operation along the spatial dimension; *f* denotes the feature expression of the intermediate information encoding operation in the horizontal and vertical directions; δ stands for the nonlinear activation function.

After batch normalization and nonlinear operations, the function is decomposed into tensors in two directions, namely $f^{h}\in R^{C/r\times H}$ and $f^{w}\in R^{C/r\times W}$. After the 1×1 convolution transformation functions $F_{h}$ and $F_{w}$ transform $f^{h}$ and $f^{w}$ into tensors with the same number of channels, respectively. Then input into *X* as shown in Equation (4):

(4) $$\begin{array}{c} \left\{\begin{array}{c} g^{h}=\delta \left(F_{h}\left(f^{h}\right)\right)\\ g^{w}=\delta \left(f_{w}\left(f^{w}\right)\right) \end{array}\right. \end{array}$$

where δ represents the sigmoid activation function, *r* is the reduction ratio of the control module that reduces the channel number of *f*, and finally the input values $x_{c}$ from each direction are re-weighted to form the final output of the CA module, as shown in Equation (5):

(5) $$\begin{array}{c} y_{c}\left(i,j\right)=x_{c}\left(i,j\right)\times g_{c}^{h}\left(i\right)\times g_{c}^{w}\left(j\right) \end{array}$$

where $g_{c}^{h}\left(i\right)$ and $g_{c}^{w}\left(j\right)$ denote the weight value of the *c*th channel in different directions respectively; $y_{c}\left(i,j\right)$ is the attention module output value of the *c*th channel.

The CA mechanism enhances the model’s ability to recognize regions of interest in images by strengthening feature expression, capturing long-range dependencies, implementing direction-aware and position-sensitive attention enhancement, and providing more precise localization capabilities. This operational method enables the CA module to more accurately locate the positions of effective features, thereby achieving precise identification of various defects on the PCB board.

#### 2.2.3. Improvements of Loss Function

In YOLOv8, when using Complete Intersection over Union (CIoU) Loss as the bounding box loss, there are indeed issues caused by the inevitable presence of low-quality data in the training data. CIoU Loss, in addition to considering IoU, also incorporates geometric measurements such as distance and aspect ratio. These geometric measurements may exacerbate the penalization for such data when faced with low-quality data, as low-quality data often implies inaccurate labeling or significant deviation from the actual situation. To address this issue, Tong et al. [22] proposed WIoU Loss (Wise-IoU) in 2023, which adopts a dynamic non-monotonic focusing mechanism and evaluates anchor box quality based on outlier degree rather than IoU. This paper employs the Weighted IoU (WIoU) Loss, which mitigates the penalization of geometric measurements when the anchor box and the target box exhibit substantial overlap, avoiding excessive intervention in the training to enable the model to have better generalization capabilities.

**Figure 4:**
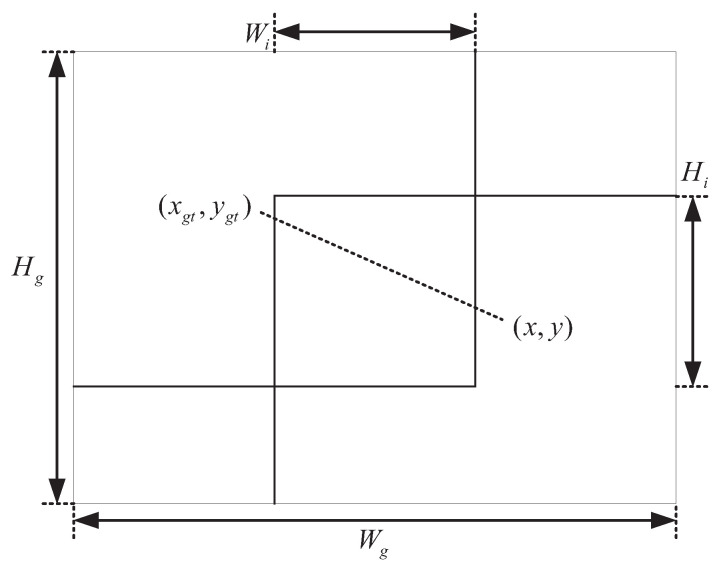
Illustration of BBox Overlap.

As shown in Figure 4, IoU is a metric for measuring the degree of overlap between predicted bounding boxes and true bounding boxes. Additionally, $L_{IoU}$ is defined as Equation (6). If there is no overlap between bounding boxes, the gradient of $L_{IoU}$ in the reverse direction disappears, which prevents the gradient from being updated during training. WIoU constructs an attention-based bounding box loss, as shown in Equation (8):

(6) $$\begin{array}{c} L_{IoU}=1-IoU=1-\frac{W_{i}H_{i}}{wh+w_{gt}h_{gt}-W_{i}H_{i}} \end{array}$$

(7) $$\begin{array}{c} R_{WIoU}=\exp\left(\frac{{\left(x-x_{gt}\right)}^{2}+{\left(y-y_{gt}\right)}^{2}}{{\left(W_{g}^{2}+H_{g}^{2}\right)}^{*}}\right) \end{array}$$

(8) $$\begin{array}{c} L_{WIoU}=R_{WioU}L_{IoU} \end{array}$$

where $R_{WIoU}\in \left[1,e\right)$ enlarge the $L_{IoU}$ of the regular anchor box. $L_{IoU}\in \left[0,1\right]$ will reduce the $R_{WIoU}$ of high-quality anchor boxes and significantly decrease the focus on the center point distance when there is good overlap between the anchor box and the target box. Separating $W_{g}$ and $H_{g}$ from the computation graph (with the superscript * indicating this operation) is to prevent $R_{WIoU}$ from generating gradients that hinder convergence.

#### 2.2.4. Adding a Small Object Detection Branch

YOLOv8 has a default architecture with three detection heads for handling multi-scale targets. Taking an input image of 640×640 pixels as an example, the feature map sizes and corresponding receptive field strides for the three detection heads are as follows:

- P3/8 corresponds to a detection feature map of size 80×80 with an effective stride of 8 pixels, primarily used for detecting medium-sized targets with dimensions of 16×16 pixels or larger.
- P4/16 corresponds to a detection feature map of size 40×40 with an effective stride of 16 pixels, used for detecting larger targets with dimensions of 32×32 pixels or larger.
- P5/32 corresponds to a feature map of size 20×20 with an effective stride of 32 pixels, mainly used for detecting large targets with dimensions of 64×64 pixels or larger.

Although the above multi-scale design improves the model’s adaptability to different scales to some extent, its detection capability for tiny targets (typically defined as those smaller than 16×16 pixels) remains insufficient, manifesting as a high miss rate and poor localization accuracy for small targets.

To enhance the model’s perception of tiny targets, this paper introduces an additional tiny target detection head (P2/4) based on the original three detection heads. This detection head corresponds to a downsampling factor of 4, with a feature map size of 160×160 and a receptive field stride of 4 pixels. Compared with the original shallowest P3 detection head (stride 8), the P2 head preserves higher-resolution spatial detail information, thereby significantly improving the feature representation capability and detection accuracy for tiny targets. By integrating the P2 branch into the multi-scale detection head architecture, the model achieves accurate detection of tiny targets without substantially increasing the computational overhead.

## 3. Data Preparation and Experimental Configuration

### 3.1. Data Preparation

**Figure 5:**
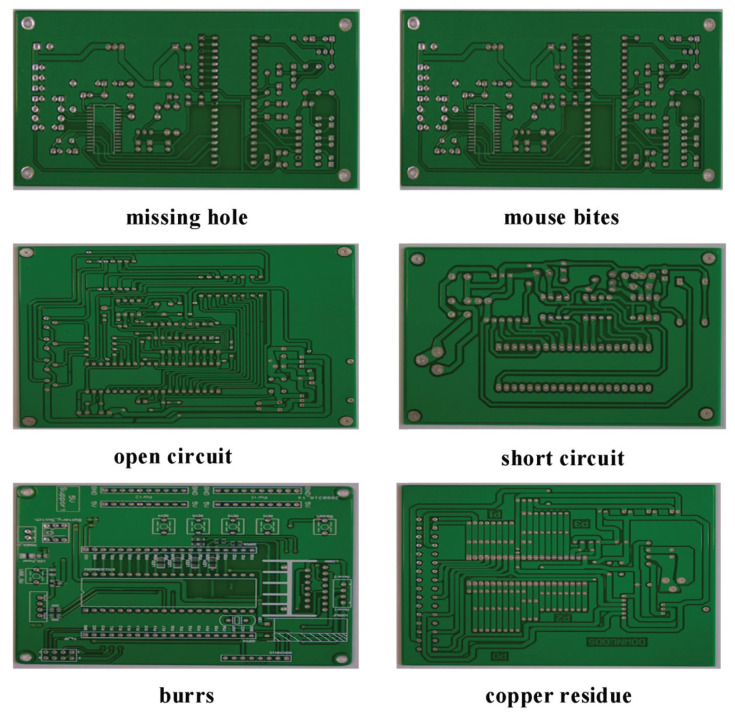
Partial Dataset.

This dataset is sourced from 693 PCB defect images released by a laboratory at Peking University, including the following types of PCB defects: short circuit, burr, miscellaneous copper, missing hole, mouse bite, and open circuit. Additionally, 6319 images were collected from actual PCB production processes, resulting in a total of 7012 images. Sample images are shown in Figure 5.

To ensure the quality of data annotation, all images were manually annotated using the LabelImg tool in the YOLO format. The annotation process was first carried out independently by two engineering technicians with experience in PCB inspection, followed by a cross-check conducted by a third senior engineer. In cases where discrepancies existed in the annotation results, the final labels were determined through manual re-examination to improve the consistency and accuracy of the data annotation.

The actual industrial dataset was collected from a PCB production inspection platform, using an industrial camera equipped with a ring LED white light source for imaging. During the acquisition process, a fixed working distance, fixed exposure time, and stable illumination conditions were maintained to reduce the impact of lighting variations on defect detection results.

**Figure 6:**
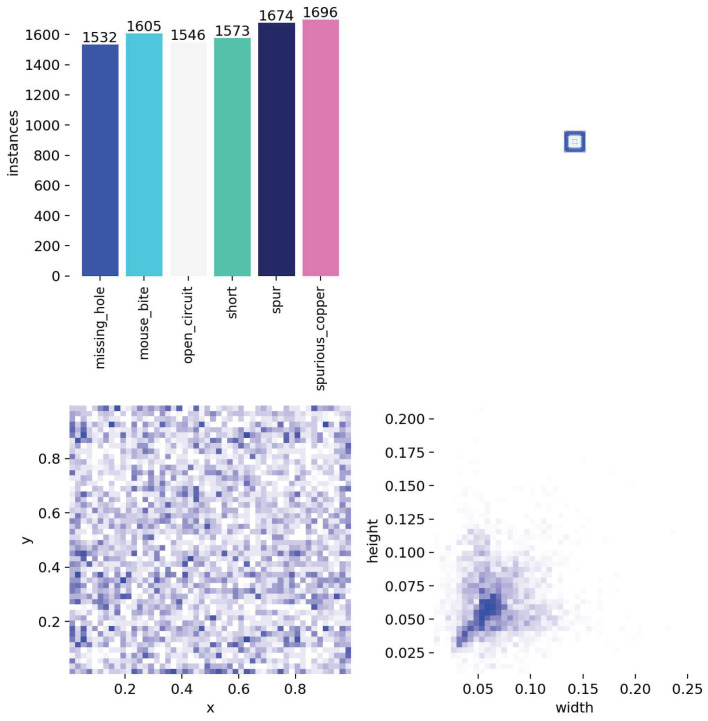
Dataset Distribution.

The training set and validation set were split in an 8:2 ratio, ensuring that the same PCB sample did not appear in both sets to prevent data leakage and to enhance the credibility and generalization ability of the experimental results. Data Availability Statement: The public PCB defect dataset used in this study is from Peking University Laboratory and can be accessed by readers at The industrial dataset collected from actual production lines cannot be publicly released at this time due to confidentiality agreements signed with the company. Researchers who wish to access this dataset may contact the corresponding author via email and, upon obtaining approval from the data owner and signing a data use agreement, will be granted access. The dataset distribution is shown in Figure 6.

The average number of samples per class ranges from 1500 to 1700, indicating a relatively balanced data distribution that avoids the potential impact of class imbalance on model training. Moreover, as shown in the distribution map of target center positions, defect instances are uniformly distributed across the entire image area, demonstrating that the training data adequately represent the spatial distribution of real-world industrial scenarios. The distribution of target width and height shows that most defects are small objects (width: 0.04–0.1, height: 0.03–0.08), further validating the applicability of the model for small-scale object detection.

### 3.2. Experimental Configuration

The experimental environment in this paper is as follows: a desktop computer with the Windows 10 operating system, an Intel(R) Core i9-13900K processor (Santa Clara, CA, USA), 16 GB of host memory, and an NVIDIA GeForce RTX 4070 (Santa Clara, CA, USA) graphics card with 12 GB of video memory. The software environment is configured with PyTorch 1.11.0 and CUDA version 11.3. The input image size is set to 640×640 pixels. Six defect categories are detected, with a batch size of 8 and a total of 200 training epochs, using the SGD optimizer. The initial learning rate is 0.01, momentum is 0.937, and weight decay is 0.0005. The data augmentation strategies include: random flipping (50% probability), HSV hue/saturation/value augmentation (±0.015), mosaic augmentation (for the first 100 epochs), and MixUp augmentation (10% probability). All models are trained under the same number of epochs and the same hardware environment to ensure fair experimental comparison. The experiments in this paper use mean average precision (mAP) to evaluate the performance of the trained PCB defect detection models. The expression of mAP and the meaning of each parameter can be found in reference [23].

## 4. Experimental Results and Analysis

**Figure 7:**
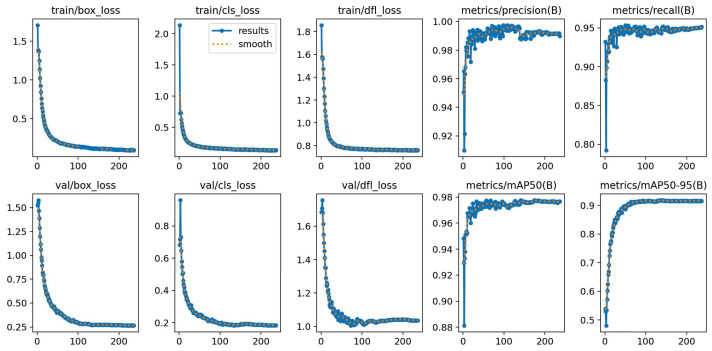
Loss Curves.

The loss curves of the improved YOLOv8 algorithm during training are shown in Figure 7. As the number of training epochs increases, the loss gradually decreases and the algorithm tends to converge. In Figure 7, the left side shows the localization loss on the training set, the middle shows the class loss, and the right side shows the distribution focal loss.

From the training curves, it can be observed that the model exhibits good convergence characteristics and stability in the task of detecting the six types of PCB defects.

In the early stage of training, the train/box_loss, train/cls_loss, and train/dfl_loss all decrease rapidly, indicating that the model can quickly learn the basic features of target localization and class discrimination. Subsequently, the loss functions enter a phase of gradual decline and stabilize after approximately 100 epochs, demonstrating that the model parameters are progressively converging.

The loss curves on the validation set are consistent with those on the training set, and the gap between them remains small, with no obvious divergence. This indicates that the model does not suffer from significant overfitting, and that the data distribution and annotation quality are relatively consistent, giving the model good generalization ability. In terms of performance metrics, mAP@0.5 reaches approximately 0.97, and mAP@0.5:0.95 reaches approximately 0.91. The model maintains strong detection capability even under high IoU criteria, demonstrating high localization accuracy and high-quality bounding box regression.

**Figure 8:**
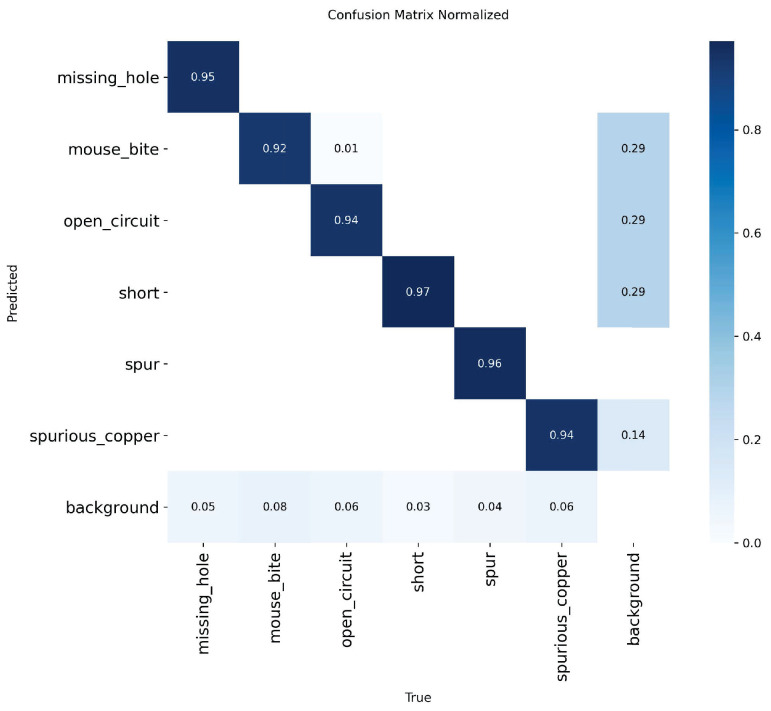
Normalized confusion matrix.

As shown in Figure 8, the normalized confusion matrix further illustrates the model’s classification capability and misclassification patterns across different categories. The values on the main diagonal are all above 0.92, indicating that the model can effectively distinguish between different defect types, even for morphologically similar defects such as short circuit and burr, where it still maintains high accuracy. The misclassification rate for the background category is low, ranging from approximately 3% to 8%, demonstrating that the model can suppress false positives in irrelevant regions and possesses strong engineering practicality. The few misclassifications between categories mainly occur between defects with similar morphology, which is an acceptable margin of error in industrial inspection.

**Figure 9:**
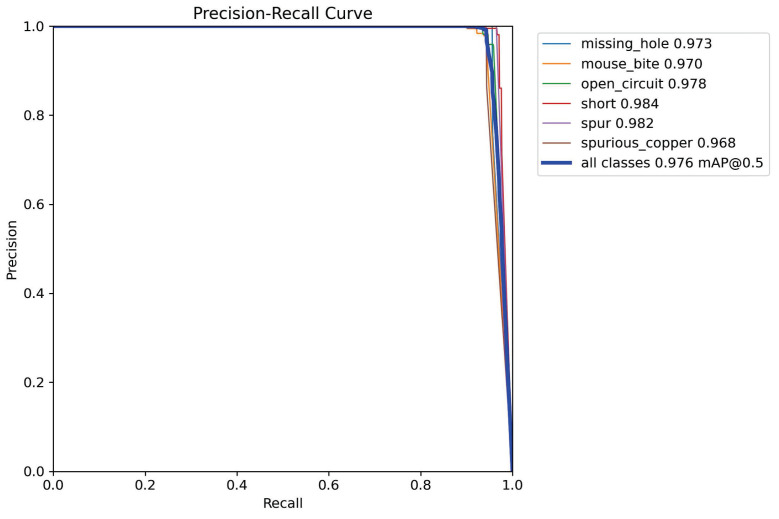
PR Curve Analysis.

Figure 9 presents the Precision–Recall curves for each category along with the overall mAP@0.5 metric. The model exhibits high precision and high recall across all defect categories, with the curves closely clustered near the top-right corner, indicating excellent performance in both reducing false positives and minimizing missed detections. The overall mAP@0.5 reaches 0.976, demonstrating that the proposed model can achieve high reliability and strong robustness in industrial PCB inspection tasks.

**Figure 10:**
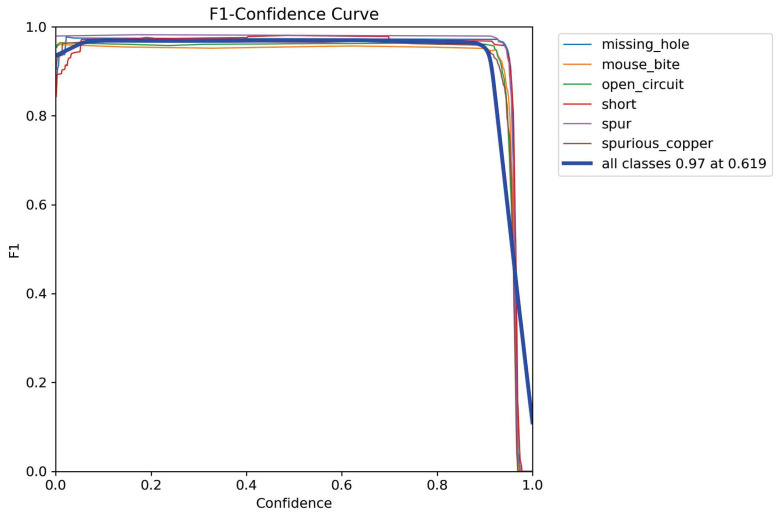
F1 Curve Analysis.

As shown in Figure 10, the F1 curve reflects the overall balance between precision and recall under different confidence thresholds. Experimental results show that the F1 score remains at a high level of 0.96–0.98 over a wide confidence interval, enabling both low false positive rate and low missed detection rate at moderate confidence thresholds. Moreover, the curve is relatively flat in the range of 0.1 to 0.9, indicating that the model is insensitive to threshold variations and possesses strong robustness, making it suitable for threshold fine-tuning according to specific requirements in practical industrial deployment.

As shown in Table 1, after replacing the Bottleneck in the original C2f module with RepGhostBottleneck, the number of parameters decreased from 9.6 M to 6.0 M, a reduction of 37.5%; FLOPs decreased from 27.2 G to 16.8 G, a reduction of 38.2%; and inference time decreased from 16.8ms to 12.4ms, a reduction of 26.2%. This indicates that RepGhostBottleneck can effectively compress model size and improve computational efficiency.

As shown in Table 2, the proposed method achieves 97.6% mAP@0.5 on the public PCB dataset, outperforming TDD-Net [12] (94.2%), Cascade R-CNN [24] (95.1%), PCB-YOLOv4 [25] (93.8%), and PCB-YOLO [26] (95.6%). In terms of the number of parameters, although the proposed method (34.0 M) has more parameters than the lightweight model PCB-YOLO (2.30 M), it achieves a 2.0 percentage point improvement in detection accuracy. Compared with traditional methods, the parameter count of the proposed method is significantly lower than that of Cascade R-CNN (68.0 M) and PCB-YOLOv4 (52.3 M). Overall, the proposed method strikes a good balance between detection accuracy and model complexity.

To further validate the effectiveness of the proposed method, this study compares its performance with that of mainstream single-stage object detection algorithms, namely YOLOv5, YOLOv7, and YOLOv8, on the task of PCB surface defect detection. Under the same AP@0.5 condition, detection accuracy was evaluated for six types of defects: missing hole, mouse bite, open circuit, short circuit, burr, and miscellaneous copper. The experimental results are shown in Table 3. The proposed method significantly outperforms the other methods in detection accuracy for each defect type.

In addition, to validate the effectiveness of the improvement strategies, this study conducted ablation experiments. The baseline model was YOLOv8, with mAP@0.5, mAP@0.5:0.95, and average detection speed used as evaluation metrics. Table 4 presents the results of the ablation experiments: compared to the original YOLOv8 network, the proposed method achieves a 6.6% improvement in mAP@0.5, an 8% improvement in mAP@0.5:0.95, and an increase in average detection speed of approximately 2 frames per second. These results demonstrate that the improvement strategies achieve significant gains in both accuracy and efficiency.

As shown in Table 4, the effectiveness of each improved module is progressively validated through ablation experiments. Baseline model: The original YOLOv8L achieves 91.0% mAP@0.5 and 83.0% mAP@0.5:0.95 on the PCB dataset, with 43.7 M parameters and an FPS of 30. Introducing RepGhostBottleneck: After replacing the Bottleneck in the C2f module with RepGhostBottleneck, the number of parameters is reduced to 27.3 M (a decrease of 37.5%), FPS increases to 36, while mAP@0.5 increases to 93.5% and mAP@0.5:0.95 increases to 86.0%. This indicates that the RepGhost module enhances feature representation capability through a feature reuse mechanism while compressing the model size. Adding the CA attention mechanism: After adding the CA coordinate attention module after the C2f layer, mAP@0.5 further improves to 94.7%, mAP@0.5:0.95 improves to 87.0%, the number of parameters increases by only 0.3 M, and FPS remains unchanged. Adding the P2 small object detection head: After adding the P2 small object detection head to the original three detection heads, mAP@0.5 reaches 97.6% and mAP@0.5:0.95 reaches 91.0%, representing improvements of 6.6 and 8.0 percentage points, respectively, compared to the baseline. Although the number of parameters increases to 34.0 M and FPS slightly decreases from 36 to 34, the detection accuracy is significantly improved, validating the effectiveness of the P2 head for small defect detection.

Overall, all improved modules have made positive contributions to the final performance. The proposed method achieves a good balance between detection accuracy (97.6% mAP@0.5) and model efficiency (34.0 M parameters, 34 FPS).

As shown in Table 5, TensorRT is used to accelerate the model inference. On an NVIDIA RTX 4070 GPU, under FP32 precision, the inference latency is 19.6 ms, the FPS is 51, the model size is 70.4 MB, and mAP@0.5 remains at 97.6%. Under FP16 precision, the inference latency decreases to 15.4 ms, the FPS increases to 65, the model size is compressed to 50.5 MB, and mAP@0.5 drops by only 0.2 percentage points. After INT8 quantization, the inference latency further decreases to 12.2 ms, the FPS reaches 82, the model size is compressed to 32.3 MB, and mAP@0.5 is 95.8%.

**Figure 11:**
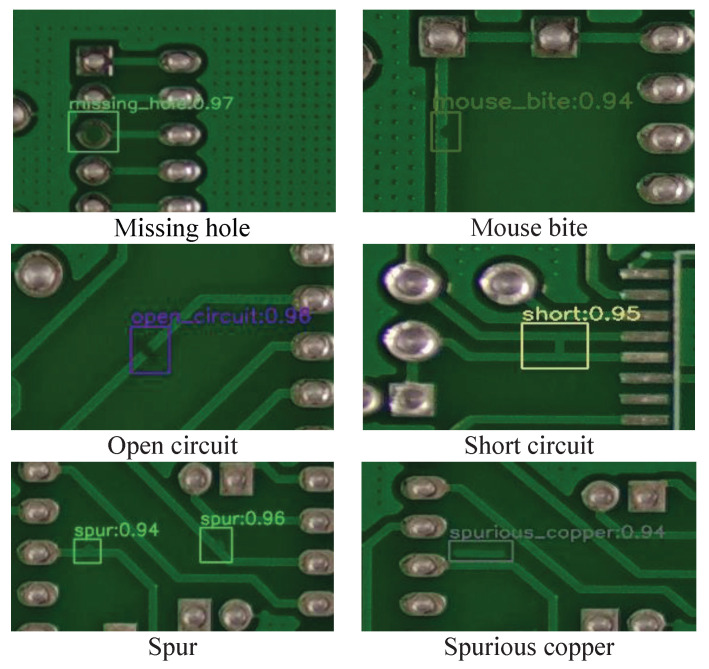
PCB Inspection Results.

Although the proposed method achieves high detection accuracy in PCB defect detection tasks, there are still some challenging detection scenarios. As shown in Figure 11, which presents some detection results of the proposed method on the PCB surface defect detection task. Six types of defects are annotated with bounding boxes in different colors: yellow for short circuits, purple for open circuits, light yellow for mouse bites, cyan for burrs, green for missing holes, and gray for stray copper. The detection performance of the model varies across different defect categories. Specifically, the detection accuracy for mouse bites (mAP = 97.0%) and burrs (mAP = 98.2%) is relatively lower than that for other categories (short circuits and open circuits both exceed 97.8%). The reasons for this are analyzed as follows:

Mouse bites: This type of defect appears as tiny hole-like depressions on the PCB substrate, typically with a diameter of less than 0.2 mm, occupying only about 10 × 10 pixels in the image. The small size results in nearly complete loss of information in deep feature maps, relying on the P2 small object detection head for recovery.

Burrs: This type of defect appears at the edges of circuit traces, having the same color as normal copper lines, with only tiny protrusions in shape. The low contrast makes it easy for the model to misclassify burrs as normal circuit edges.

Nevertheless, the AP values of the model for both types of defects still exceed 97%, indicating that the proposed CA attention and P2 head mechanisms effectively alleviate the above issues.

Limitations: It should be noted that the proposed method has currently only been evaluated on a single combined dataset (public dataset + industrial dataset collected from the same production line), where the lighting conditions, imaging parameters, and defect distributions are relatively fixed. Therefore, the cross-dataset generalization capability of the model under different PCB production lines, different lighting conditions, and different imaging devices has not yet been validated. This is the main limitation of the proposed method at present.

Overall, the proposed method can effectively improve the detection performance of common PCB defects. However, there remains room for further optimization for targets with extreme aspect ratios and defects under complex lighting conditions. In the future, we will consider introducing rotated object detection, Transformer architectures, and defect segmentation methods to further enhance the robustness and generalization capability of the model.

In addition, this method has not been specifically optimized for elongated defects (e.g., long scratches and long cracks with an aspect ratio greater than 10:1). The anchor box mechanism of the YOLO series algorithms is based on preset aspect ratios and responds inadequately to targets with extreme aspect ratios. In future work, we plan to introduce Deformable Convolutional Networks (DCNs) or strip attention mechanisms to enhance the feature extraction capability for slender defects.

## 5. Conclusions

This paper proposes a PCB surface defect detection method based on an improved YOLOv8 architecture. Compared with the original YOLOv8L baseline, this method achieves a relatively lightweight design with a reduction in the number of parameters by approximately 22%. It should be noted that the term “lightweight” in this paper is relative to the chosen baseline. Compared with extremely lightweight detectors, the proposed model places greater emphasis on the balance between detection accuracy and efficiency. To address the challenges of small defect scales, high detection difficulty, and strict requirements for industrial deployment efficiency, RepGhostBottleneck is introduced to construct a lightweight backbone network. Furthermore, YOLOv8 is enhanced by incorporating the Coordinate Attention mechanism, a P2 small target detection head, and the WIoU loss function.

Experimental results show that the proposed method achieves 97.6% mAP@0.5 and 91% mAP@0.5:0.95 on the constructed dataset, demonstrating significant improvements in both detection accuracy and inference efficiency compared to the original YOLOv8 model. Moreover, the method can effectively detect various PCB surface defects, including missing holes, mouse bites, open circuits, short circuits, spurs, and spurious copper, indicating strong potential for industrial application.

This paper proposes a PCB surface defect detection method based on an improved YOLOv8 architecture. Compared with the original YOLOv8L baseline, this method achieves a relatively lightweight design with a reduction in the number of parameters by approximately 22%. It should be noted that the term “lightweight” in this paper is relative to the chosen baseline. Compared with extremely lightweight detectors, the proposed model places greater emphasis on the balance between detection accuracy and efficiency. Future work will focus on Transformer-based feature extraction methods, rotated object detection techniques, and deployment optimization on edge computing devices to further enhance the applicability of the model in real industrial environments.

## Figures and Tables

**Table 1:** Comparison of model complexity before and after replacing with RepGhostBottleneck (%).

Module	Number of Parameters	FLOPs (G)	Inference Time (ms)
Original C2f Bottleneck	9.6	27.2	16.8
RepGhostBottleneck	6.0	16.8	12.4
Reduction ratio	37.5%	38.2%	26.2%

**Table 2:** Comparison with existing PCB-dedicated detection methods.

Methods	Year	mAP@0.5	Number of Parameters	FPS
TDD-Net	2019	94.2	14.5	18
Cascade R-CNN	2023	95.1	68.0	8
PCB-YOLOv4	2021	93.8	52.3	22
PCB-YOLO	2026	95.6	2.3	160
Ours	2026	97.6	34.0	34

**Table 3:** Comparison of Average Precision (AP) for PCB Surface Defect Detection using Different Models (%).

Network Model	Missing Holes	Mouse Bites	Open Circuits	Short Circuits	Burrs	Miscellaneous Copper
YOLOv5	91.6	85.7	90.4	91.2	90.3	87.6
YOLOv7	95.9	86.9	93.5	92.1	90.6	92.5
YOLOv8	97.2	91.9	96.0	93.8	93.1	96.3
Ours	97.3	97.0	97.8	98.4	98.2	96.8

**Table 4:** Ablation Study.

YOLOv8	RepGhost	CA	WIoU	P2 Head	mAP@50	mAP @50:95	FPS	Number of Parameters (M)
✓					0.91	0.83	30	43.7
✓	✓				0.935	0.86	36	27.3
✓	✓	✓			0.947	0.87	36	27.6
✓	✓	✓	✓		0.952	0.89	36	27.6
✓	✓		✓	✓	0.976	0.91	34	34.0

**Table 5:** Performance Evaluation.

Precision Mode	Inference Latency	FPS	Model Size (MB)	mAP@0.5
FP32	19.6	51	70.4	97.6
FP16	15.4	65	50.5	97.4
INT8	12.2	82	32.3	95.8

## Data Availability

The original contributions presented in this study are included in the article. Further inquiries can be directed to the corresponding author.
